# Maladaptive behaviours in adolescence and their associations with personality traits, emotion dysregulation and other clinical features in a sample of Italian students: a cross-sectional study

**DOI:** 10.1186/s40479-021-00154-w

**Published:** 2021-05-04

**Authors:** Mariangela Lanfredi, Ambra Macis, Clarissa Ferrari, Serena Meloni, Laura Pedrini, Maria Elena Ridolfi, Valentina Zonca, Nadia Cattane, Anna Cattaneo, Roberta Rossi

**Affiliations:** 1 Unit of Psychiatry, IRCCS Istituto Centro San Giovanni di Dio Fatebenefratelli, via Pilastroni 4, I-25125 Brescia, Italy; 2 Service of Statistics, IRCCS Istituto Centro San Giovanni di Dio Fatebenefratelli, Brescia, Italy; 3 Outpatient Psychiatry Services, CSM Fano AV1, Fano, Marche Italy; 4 Biological Psychiatry Unit, IRCCS Istituto Centro San Giovanni di Dio Fatebenefratelli, Brescia, Italy; 5 Stress, Psychiatry and Immunology Laboratory, Department of Psychological Medicine, Institute of Psychiatry, Psychology and Neuroscience, King’s College, London, UK; 6 Department of Pharmacological Biomolecular Sciences, University of Milan, Milano, Italy

**Keywords:** Maladaptive behaviours, Emotion dysregulation, Borderline personality disorder, Depression, Adolescents

## Abstract

**Background:**

Emotion Dysregulation (ED), childhood trauma and personality are linked to the occurrence of maladaptive behaviours in adolescence which, in turn, may be related to increased risk for psychopathology in the life course. We sought to explore the relationship among the occurrence of different clusters of maladaptive behaviours and ED, clinical features (i.e. impulsivity, childhood maltreatment, anxiety, depressive symptoms) and personality traits that have been found to be associated to Borderline Personality Disorder (BPD), in a sample of 179 adolescent students.

**Methods:**

Multiple Correspondence Analysis (MCA) was applied to detect clustered types of maladaptive behaviours and groups of students were defined as individuals engaging in these clustered behaviours (non-suicidal self-injury-NSSI, binge eating, binge drinking, cannabis use, and sexual risk behaviours). Logistic models were used to evaluate the association among clinical scales, and student groups. Mediation analysis was used to evaluate whether clinical features affected the association between personality traits and student groups.

**Results:**

MCA analysis allowed to identify three student groups: NSSI/binge eating (NSSI-BE) behaviours, other maladaptive behaviours and “none”. Higher scores in ED, impulsivity, childhood maltreatment, anxiety and depressive symptoms increased the risk of belonging to the cluster of NSSI-BE behaviours compared to the other two groups. ED, depression and anxiety symptoms were found to be mediators of the relationship between specific personality traits, mainly pertaining to the negative affectivity construct, and NSSI/BE.

**Conclusions:**

Individuals engaging in NSSI-BE behaviours represent a vulnerable adolescent population. ED, depression and anxiety were mediators of the relationship between a variety of personality traits related to BPD and NSSI and binge eating behaviours. Findings have important clinical implications in terms of prevention and interventions among adolescents engaging in self-damaging behaviours.

**Supplementary Information:**

The online version contains supplementary material available at 10.1186/s40479-021-00154-w.

## Background

Adolescence represents a sensitive and vulnerable period for the development of internalising and externalising symptoms [1] and of a wide range of problematic behaviours, often persisting into adulthood [2]. Although problematic behaviours may occur within the framework of a normal development in adolescence, their recurrence could represent a risk factor for developing mental health problems at an older age [3]. Emotion Dysregulation (ED) is a multifaceted construct involving different components: a lack of awareness, understanding, and acceptance of emotions; an inability to control behaviours during an emotional distress; lack of access to adaptive strategies for modulating the duration and/or intensity of aversive emotional experiences; and an unwillingness to experience emotional distress [4]. A growing body of research indicates that heightened ED may increase the likelihood of engaging in maladaptive behaviours including Non-Suicidal Self-Injury (NSSI), unsafe sex, aggressive behaviours, substance use, and disordered eating [5, 6]. NSSI in adolescence is a serious health concern since it is a risk behavioural marker for the incidence of mental illness in general [7] and repetitive NSSI represents a predictive factor for progression to suicidal ideation or suicide attempts [8]. NSSI is relatively common in clinical settings [9, 10] with a lifetime prevalence rate of adolescent displaying self-harm behaviours that ranges from 13 to 23%. Furthermore, in non-clinical populations, approximately 4% of individuals reported a history of self-injury [11]. A recent retrospective study among adolescents who underwent child psychiatric consultation at an Italian paediatric emergency department found that about half of those hospitalized for suicidal behaviour or suicidal ideation reported a current or lifelong history of NSSI [8, 12]. Some studies underlined the key role of adverse childhood experience in the cascade of factors that leads to maladaptive behaviours. In the complex relationship between childhood maltreatment and maladaptive behaviours, ED seems to be determining. Findings from Arens and colleagues [13] among college students showed that a history of trauma experience leads to ED, which leads to impulsivity under extreme affect (urgency) as a means of coping, and this in turn, increase the likelihood of engaging in health-risk behaviours in an attempt to quickly reduce intense negative affects. Another recent study conducted among individuals who experienced childhood adversity showed that only ED and not impulsivity mediated the relation between childhood adversities and maladaptive behaviours, alcohol-related consequences, and risky sexual behaviours [14].

According to the biosocial model, temperamental vulnerability to ED becomes a core feature of externalizing problems and internalizing problems and places adolescents and young individuals at risk for more serious forms of psychopathology [3]. Personality traits were found associated also to problematic behaviours [15]. In particular, recent studies have demonstrated that individuals engaging in NSSI show higher scores on the personality dimension of Neuroticism, and lower scores on Agreeableness and Conscientiousness dimensions than those without NSSI [16–20]. NSSI, ED, impulsive behaviours and the presence of childhood traumatic experiences are key features of Borderline Personality Disorder (BPD) and might be considered potential risk factors for the development of the disorder and might be a possible target of preventative interventions. Indeed, individuals who engage in NSSI were found to be more likely to have a cluster B personality disorder [21]. Among the specific diagnoses comprising cluster B, investigations show that BPD is associated with heightened risk for a variety of self-damaging behaviours [22]. Furthermore, recent literature indicates that ED is an important transdiagnostic process [23], affecting BPD and eating disorders more than other conditions [24]. A recent longitudinal study [25] found that adolescents engaging particularly in self-injurious behaviours and risky alcohol use represent a specific high-risk group for the development of BPD.

The present study aimed to clarify the association among maladaptive behaviours, clinical dimensions and personality traits. Firstly, we sought to describe the presence of maladaptive behaviours (i.e. NSSI, binge eating, binge drinking, cannabis use, and risky sexual behaviours) in a sample of Italian community-dwelling students and to define different groups of adolescents, based on their engaging in types of maladaptive behaviours. Considering that it is well-established that NSSI and eating disordered behaviours frequently co-occur [26] we expected that in our sample NSSI and binge eating were distinguished by other types of maladaptive behaviours. Secondly, we aimed to evaluate the associations between adolescent groups engaging in different clusters of maladaptive behaviours and ED, depression, anxiety, impulsivity, trauma experiences and BPD related personality traits. In detail, we focused on exploring the putative impact of the clinical features on the relationship between BPD-related traits and the different clusters of maladaptive behaviours. We hypothesized that individuals with higher BPD related traits were more associated to NSSI and binge eating than other types of behaviours, and that these associations may be mediated by clinical features.

## Methods

### Design

This is a cross-sectional observational study on ED and maladaptive behaviours among adolescent students that was conducted in 2018 at the IRCCS Centro San Giovanni di Dio Fatebenefratelli in Brescia (Italy)*.* The study was approved by the Local Ethical Committee (n 113/2017).

### Participants

Four high schools were invited to participate based on a well-established relationship developed during previous collaborations between the Center and the schools. The principal of each school selected the classes on the basis of organization set-up and was not influenced by the study investigators in any way. Inclusion criteria were: 1. attending one of the last two grades (4th, 5th) of upper secondary school, 2. being able to understand Italian language, 3. ability to give an informed consent. Exclusion criteria: mild or severe cognitive impairment. The students of the 4th and 5th year of upper secondary school who agreed to participate in the study signed an informed consent (or parents did, in the case of a minor). The convenience sample included 9 classes (5 classes of the 4th year and 3 classes of the 5th year) from 4 schools located in Brescia. The participating schools were 4 State upper secondary schools (2 human sciences lyceums (6 classes); 1 sciences lyceum (1 class) and a publicly subsidized upper secondary school (2 classes from a professional institute oriented on social sciences). Questionnaires were administered anonymously during class time by two researchers and students had approximately 60 min to complete them. After the assessment completion all classes received a two-session psycho-educational intervention focused on ED and impulsive behaviours in adolescence. The psycho-educational sessions (2 h each) were conducted by two clinical psychologists in usual classroom settings and during school hours.

### Measures

Participants underwent a comprehensive assessment including the following measures:

- Difficulties in Emotion Regulation Scale (DERS) [4]. The DERS is a 36-item scale on a 5-point Likert scale assessing emotion dysregulation. For our study, we used the total score with higher score indicating higher difficulties in ED.
- Personality Inventory for DSM-5 (PID-5) [27]. The PID-5 is a 220-item self-report-questionnaire measuring the Criterion B of the AMPD on a 4-point Likert scale. It includes 25 trait facets assessing the 25 maladaptive personality traits listed in DSM-5 AMPD [28], and 5 higher-order trait domains. For our analyses we exclusively selected the 7 facets that were specified as DSM-5 Section III BPD trait profile (anxiousness, depressivity, emotional lability, hostility, impulsivity, risk taking, and separation anxiety). In addition, we included 3 facets that previous studies [29–31] have found to discriminate individuals with BPD from individual with other PDs or no PDs diagnosis (i.e. suspiciousness, distractibility and perceptual dysregulation). In the present study, the mean score of each trait facet was calculated.
- Barratt Impulsiveness Scale-11 (BIS-11) [32]. The BIS is a 30-items self-report measure of impulsiveness with responses rated on a 4-point Likert scale. For the present study, we used the BIS-11 total score with higher score indicating higher impulsivity.
- Patient Health Questionnaire (PHQ-9) [33]. The PHQ-9 is a 9-item self-reported instrument assessing depressive symptom severity over the previous 2 weeks from administration.
- Screen for Child Anxiety Related Emotional Disorders (SCARE) [34]. The SCARE is a self-report 38-items scale assessing child and adolescent anxiety symptoms on a 3-point Likert scale. In the current study, total score was used as a measure of anxiety symptom severity.
- Childhood Trauma Questionnaire Short Form (CTQ SF) [35]. The CTQ is a 28-item retrospective measure of child maltreatment experiences rated on a 5-point Likert scale. For our study, we used the total score as a measure of maltreatment severity.
- Emotion Regulation Questionnaire (ERQ) [36]. The ERQ is a 10-item scale which measures two different emotion regulation strategies, Cognitive Reappraisal (CR) and Expressive Suppression (ES), by using a 7-point Likert scale. The total score for each emotion regulation strategy was calculated. The higher the score the greater the use of the emotion regulation strategy.
- Furthermore, we also collected socio-demographics variables and a checklist including yes/no questions exploring lifetime history of a variety of maladaptive behaviours (i.e. NSSI, binge eating, binge drinking, cannabis use, and risky sexual behaviours) or being victims of bullying.

### Statistical analyses

Absolute frequencies and the description of the presence of multiple behaviours across students were represented by using a Venn diagram. Subsequently, Multiple Correspondence Analysis (MCA) [37] was performed to analyse the association among maladaptive behaviours. For this purpose, lifetime history of: NSSI, binge eating, binge drinking and unprotected sexual intercourse were dichotomized as “Yes” if they occurred at least once or twice and as “No” if otherwise. Similarly, cannabis use dichotomized as “Yes” if this behaviour occurred at least three times lifetime and “No” if never occurred. The outcome of this method was represented in a two-dimensional space plot (Biplot) showing the relationships among categories. Variable categories that are in the same quadrant or that are close enough to each other suggest an association [38]. Possible clusters (defined by geometrical closeness in the Biplot) of behaviour categories were used to split the sample in homogeneous student groups which exhibited such behaviour categories.

Comparison of categorical variables was performed by using the Chi-Square test. Clinical scales and personality traits were compared across groups by using ANOVA or Kruskal-Wallis tests. Post-hoc comparisons were adjusted by using Bonferroni correction.

The association between the groups of students (defined by MCA technique described above) and the clinical scales and personality traits was evaluated through the use of univariate logistic models with group as dependent variable and clinical scales and personality traits as independent ones. Odds Ratios (ORs) were used to evaluate the strength of the association. Correlations between the clinical scales and the traits were evaluated by using the Spearman coefficient *ρ*.

Finally, any potential mediator effect of clinical scale on the group-trait relations was evaluated following the Baron and Kenny’s procedure (see Supplementary material –Methods-) performed by the Structural Equation Model -SEM- approach in order to model the variance-covariance structure of the variables involved in the mediation models. To summarize all the SEM finding and for improving the readability of any mediation effect, the outputs of the mediation models were reported in terms of associations between personality traits and the student groups evaluated also in a multiple logistic model setting, by adjusting for the clinical scales.

All tests were two-tailed, and the probability of a type I error was set at *p* < .05. The descriptive analyses were performed using IBM SPSS Statistics for Windows, Version 26.0. Armonk, NY: IBM Corp. The multivariate MCA technique, the correlations and logistic models were carried out by software R (R Core Team, 2020, version 3.6.3; with package *FactoMineR* for MCA).

## Results

### Sample characteristics

**Fig. 1:**
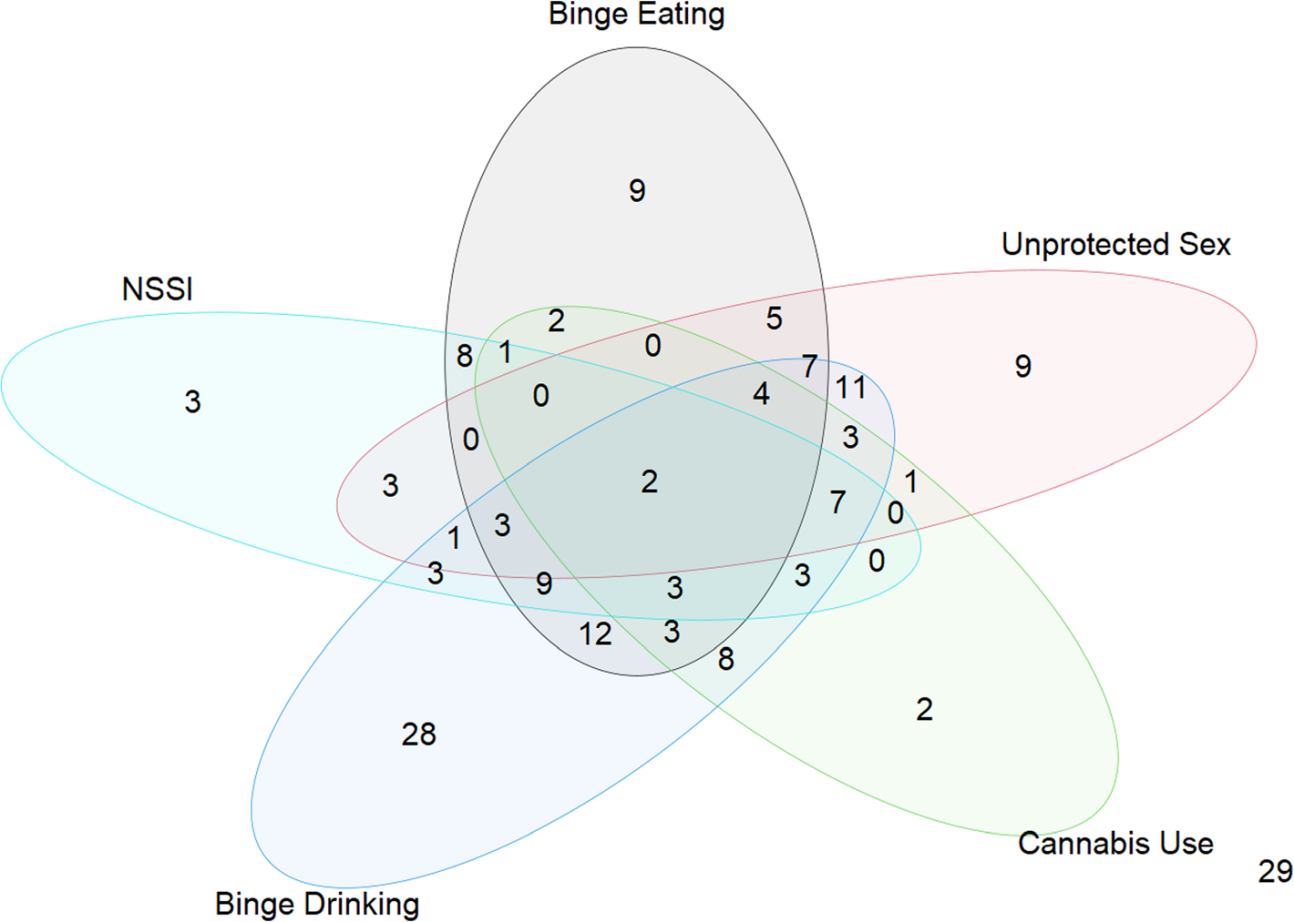
Venn diagram representing the frequency and the presence of multiple maladaptive behaviours in the sample

The overall sample was composed of a total of 179 students (82% females). Out of the 179 participants, 46 (25.7%) reported lifetime NSSI engagement, 68 (38.0%) binge eating episodes, 107 (59.8%) binge drinking behaviours, 56 (31.3%) practiced unsafe sex and 39 (21.8%) reported intake of cannabis. The frequency as well as the presence of multiple maladaptive behaviours in the student sample is represented in Fig. 1. Twenty-nine students (16.0%) had no maladaptive behaviours, 51 students (29.0%) enacted only one maladaptive behaviour (3 engaged only in NSSI, 9 in binge-eating, 9 in risky sexual behaviour, 2 in cannabis use and 28 in binge-drinking), while the remaining 99 students (55.0%) had more than one maladaptive behaviour. Correlations of all the clinical scales and personality traits were evaluated (Supplementary materials, Additional Fig. 1).

### Association between types of maladaptive behaviours

**Fig. 2:**
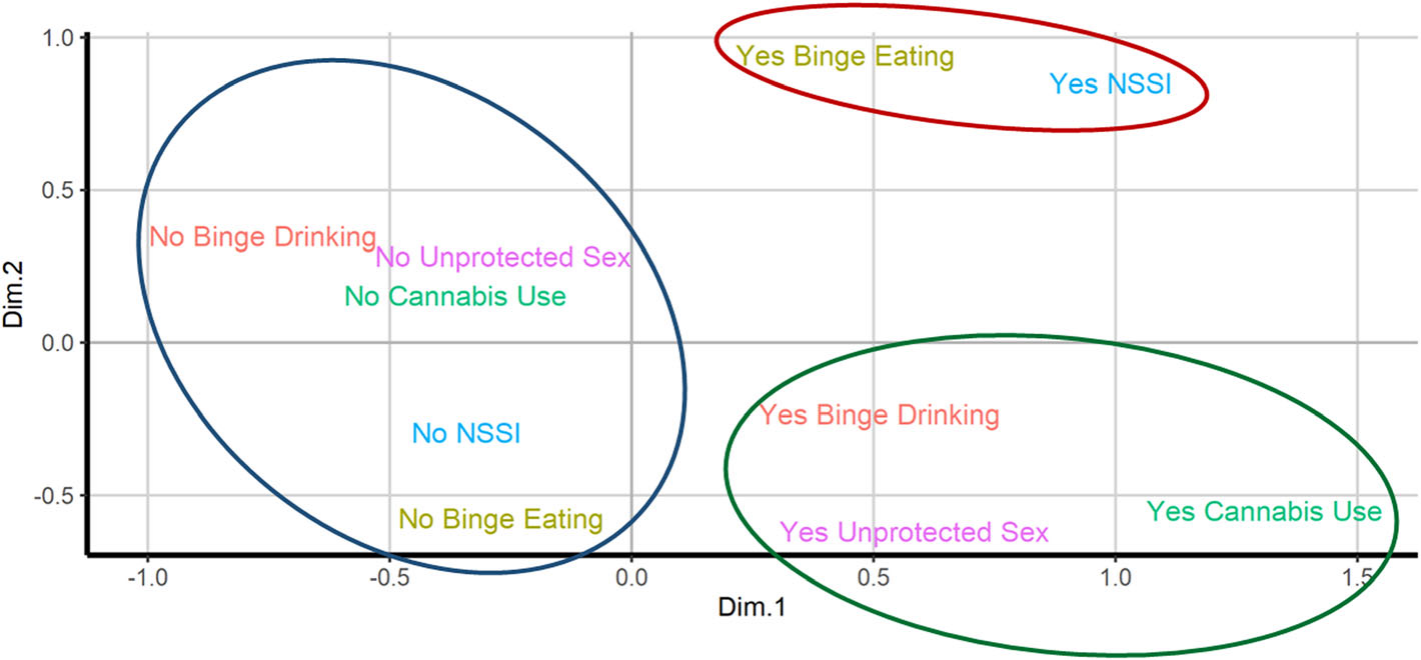
Biplot of results obtained through Multiple Correspondence Analysis

MCA data-driven technique was performed to detect which behaviours were associated each other and clustered, as the majority of the students presented more than one maladaptive behaviour. The MCA results are shown through the Biplot representation (Fig. 2). A first distinction is showed between the left and right side of the Biplot: the absence of maladaptive behaviours (all “No” categories) is displayed in the left side of the Figure (blue circle) whereas all the “Yes” categories are in the right side. In addition, among “Yes” categories, two clusters appeared: red circle, on the top right of the plot, including NSSI and binge-eating and green circle (on the bottom right) including binge-drinking, using cannabis and having unprotected sex. Through the MCA data-driven technique, thus, three student groups were identified on the basis of the closeness of the categories (cluster represented by circles in Fig. 2): i) a first group composed by those students who didn’t engage in any maladaptive behaviour (*n* = 29, 16.0%), hereafter NONE group; ii) a second group of students who engaged in at least one behaviour between NSSI or binge-eating, independently of the other maladaptive behaviours (*n* = 88, 49%), hereafter NSSI-BE group; and iii) a third group including the remaining students (*n* = 62, 35.0%, i.e. students who engaged in any other maladaptive behaviour, one or more, except for NSSI and binge-eating), hereafter OTHER group.

### Descriptive statistics and association analysis between clusters of maladaptive behaviours and clinical scales

The three student groups were not significantly different for sex (*p* = .146) and age (*p* = .433) thus no further adjustment for these variables was performed in the subsequent analyses. The NSSI-BE group exhibited higher mean scores in all the clinical scales and in all the BPD-related traits (Table 1). Moreover, the majority of the adolescents in the NSSI-BE group (56.3%) was victimized by bulling over their life-course: this percentage was higher than those observed in the other two groups. Interestingly, the post-hoc comparisons showed that the NONE and OTHER groups were not significantly different from each other, but they both significantly differed from the NSSI-BE group. For this reason, we decided to merge these two groups together in the NO-NSSI-BE group.

**Table 1:** Clinical characteristics of the overall sample divided in three groups

	NONE (0) *N* = 29	NSSI-BE (1) *N* = 88	OTHER (2) *N* = 62	*p*-value	Post-hoc
	Mean (SD)	Mean (SD)	Mean (SD)		
Sex, *n* (%)					
M	4 (13.8)	11 (12.6)	15 (25.0)	146	
F	25 (86.2)	76 (87.4)	45 (75.0)		
Age	17.8 (1.0)	17.8 (0.8)	18.0 (0.9)	433	
DERS	77.5 (16.2)	97.5 (26.2)	79.0 (19.5)	**<.001**	(0) vs (1) **.001**
					(1) vs (2) **< .001**
CTQ	33.9 (11.0)	37.1 (13.1)	32.6 (7.7)	**.014**	(1) vs (2) **.020**
BIS-11	58.5 (9.0)	64.1 (9.6)	60.3 (11.0)	**.011**	(0) vs (1) **.030**
PHQ-9	5.5 (2.3)	10.9 (5.5)	6.6 (4.0)	<.001	(0) vs (1) < .001
					(1) vs (2) < .001
SCARE	62.2 (9.4)	69.6 (13.0)	61.7 (10.3)	**<.001**	(0) vs (1) **.032**
					(1) vs (2) **.001**
ERQ-CR	5.0 (1.1)	4.6 (1.1)	4.9 (0.9)	.074	
ERQ-ES	3.3 (1.2)	3.8 (1.1)	3.5 (1.2)	.106	
Separation Anxiety	0.9 (0.7)	1.4 (0.7)	1.1 (0.6)	**<.001**	(0) vs (1) **.001**
					(1) vs (2) **.013**
Anxiousness	1.4 (0.6)	1.8 (0.7)	1.4 (0.6)	**<.001**	(0) vs (1) **.019**
					(1) vs (2) **< .001**
Depressivity	0.7 (0.4)	1.0 (0.6)	0.7 (0.5)	**.001**	(0) vs (1) **.013**
					(1) vs (2) **.005**
Impulsivity	0.8 (0.5)	1.1 (0.7)	0.9 (0.6)	**.044**	(0) vs (1) .063
Emotional lability	1.3 (0.7)	1.8 (0.8)	1.3 (0.7)	**<.001**	(0) vs (1) **.001**
					(1) vs (2) **< .001**
Hostility	1.0 (0.5)	1.3 (0.5)	1.0 (0.5)	**.002**	(0) vs (1) **.006**
					(1) vs (2) **.042**
Risk taking	1.0 (0.4)	1.1 (0.5)	1.2 (0.5)	.250	
Perceptual dysregulation	0.5 (0.4)	0.8 (0.5)	0.6 (0.9)	**<.001**	(0) vs (1) **.009**
					(1) vs (2) **< .001**
Distractibility	0.8 (0.6)	1.3 (0.6)	0.9 (0.6)	**<.001**	(0) vs (1) **.002**
					(1) vs (2) **< .001**
Suspiciousness	1.2 (0.5)	1.4 (0.6)	1.3 (0.5)	.162	
Victimization by bulling, *n* (%)					
Yes	9 (31.0)	49 (56.3)	17 (27.9)	**.001**	
No	20 (69.0)	38 (43.7)	44 (72.1)		

*DERS* Difficulties in Emotion Regulation Scale, *CTQ* Childhood Trauma Questionnaire, *BIS* Barratt Impulsiveness Scale, PHQ-9 Patient Health Questionnaire, *SCARE* Screen for Child Anxiety Related Emotional Disorders, *ERQ* Emotion Regulation Questionnaire (−CR Cognitive Reappraisal; −ES Expressive Suppression)

Kruskal-Wallis for DERS total score, CTQ total score, PHQ-9 total score, SCARE total score, Emotional lability, Separation anxiety, Depressivity, Impulsivity, Risk taking, Hostility, Suspiciousness, Distractibility and Perceptual dysregulation

ANOVA for Age, BIS-11 total score and Anxiousness

Chi-Square test for Sex and Victimization by bulling

Univariate logistic models were then performed to evaluate the strength of the association between the clinical scales (those resulted significantly associated are shown in Table 1) and the new group variable with categories NSSI-BE vs NO-NSSI-BE (Table 2). All the examined clinical scales were significantly associated with the groups, with ORs which are all larger than 1. This association resulted particularly strong in the PHQ-9 and DERS scales where, with an increase of 1 standard deviation (SD) of the score, the probability of belonging to NSSI-BE group was about 3 and 2.5 times higher for PHQ-9 and DERS, respectively.

**Table 2:** Results of univariate logistic models. Association between the clinical tools and the groups (NSSI-BE vs NO-NSSI-BE)

Independent variables	OR ^#^	*p*-value	AIC
PHQ-9	3.09	**<.001**	209.64
SCARE	2.04	**<.001**	215.67
DERS	2.50	**<.001**	222.92
BIS-11	1.58	**.005**	240.76
CTQ	1.56	**.021**	245.63

*PHQ-9* Patient Health Questionnaire, *SCARE* Screen for Child Anxiety Related Emotional Disorders, *DERS* Difficulties in Emotion Regulation Scale, *BIS* Barratt Impulsiveness Scale, *CTQ* Childhood Trauma Questionnaire

^#^Odds Ratio evaluated on the standardized values. The reference group is NO-NSSI-BE

### Mediation role of clinical features on the association between clusters of maladaptive behaviours and personality traits

Analysis ascertained that all clinical scales were strongly associated with maladaptive behaviours, hence our attention to personality traits. In particular, we sought to evaluate any association of significant (see Table 1) personality traits with the two-group variable and, in addition, to measure the effect of clinical scales on such associations.

All the traits were significantly associated with the group variable (first column of Table 3 -first step of Baron and Kenny’s procedure-) with the larger effect for emotional lability and distractibility (OR equal to 2.13 and 2.29 respectively). Moreover, with respect to significant traits, all the five clinical scales (except for CTQ in separation anxiety) showed moderate/high correlations within each trait (second column of Table 3 -second step of Baron and Kenny’s procedure-). This led us to hypothesize potential mediation effects (of clinical scales on the relation personality trait-group) that were assessed through path diagrams performed by the Structural Equation Model -SEM- approach. To summarize all the SEM finding and to show how the clinical scales can affect the direct effects of personality traits on the group variable, the results of the mediation models are reported in terms of: i) OR adjusted for the effect of clinical scales, ii) significance of clinical scale in explain the dependent group variable when added in the multiple logistic model, iii) goodness of fit -by Akaike Information Criteria (AIC) index- of the logistic model (last three columns of Table 3). Interestingly, the adjustment for DERS and PHQ-9 affected the association with the group variable for emotional lability and hostility by significantly reducing the OR from 2.13 and 1.66 (of the unadjusted models for emotional lability and hostility) to 1.43 (for emotional lability) and 1.27 and 1.23 (for hostility). Moreover, these clinical scales remained significant in the multiple model with emotional lability (*p* = .003, *p* < .001) and in the model with hostility (*p* < .001 for both scales). The AIC values markedly decreased when these variables were individually added (for example, for emotional lability: from 229.9 of the unadjusted model to 222 or 208 of the adjusted models), showing that the goodness of fit improved when each of DERS or PHQ-9 scale were added to the multiple model together with trait. In other words, DERS and PHQ-9 scales affected the relation group-trait making it no longer significant. Then, considering the significant association of these scales with the group variable (Table 2) and the significant correlations between traits and scales, the hypothesis of DERS and PHQ-9 as mediators of the relation trait-maladaptive behaviours was confirmed. Similar results were found for anxiousness, depressivity, impulsivity, and perceptual dysregulation in which the SCARE scale was found as mediator variable along with DERS and PHQ-9. Differently, for separation anxiety and distractibility the adjustment for the clinical scales did not affect the relation group-trait.

**Table 3:** Results of mediation models reported in terms of the association among the personality traits, clinical scales and the student group variable (NSSI-BE vs NO-NSSI-BE)

	Unadjusted results		Adjusted (for clinical scale) results		
BPD traits	Unadjusted^#^ OR (*p*-value)	Spearman *ρ* (*p*-value)	Adjusted^#^ OR (*p*-value)	Clinical scale *p*-value	AIC
**Separation Anxiety**	**1.91 (<.001)**				235.50
DERS		0.40 (<.001)	**1.51 (.022)**	**<.001**	219.45
CTQ		0.08 (.289)	**1.90 (<.001)**	**.028**	231.68
BIS-11		0.23 (.002)	**1.75 (.001)**	**.041**	231.69
SCARE		0.33 (<.001)	**1.56 (.014)**	**.002**	211.36
PHQ-9		0.32 (<.001)	**1.52 (.021)**	**<.001**	206.14
**Anxiousness**	**1.97 (<.001)**				234.08
DERS*		0.67 (<.001)	1.23 (.329)	**.001**	223.96
CTQ		0.38 (<.001)	**1.84 (.001)**	.273	234.77
BIS-11		0.24 (.001)	**1.82 (.001)**	**.026**	229.62
SCARE*		0.73 (<.001)	1.38 (.181)	**.054**	215.85
PHQ-9*		0.68 (<.001)	1.13 (.560)	**<.001**	211.30
**Depressivity**	**1.98 (<.001)**				234.48
DERS*		0.58 (<.001)	1.30 (.219)	**.001**	223.39
CTQ		0.44 (<.001)	**1.85 (.001)**	.237	234.92
BIS-11		0.23 (.002)	**1.81 (.001)**	**.030**	230.40
SCARE*		0.46 (<.001)	1.45 (.058)	**.004**	213.92
PHQ-9*		0.62 (<.001)	1.05 (.833)	**<.001**	211.59
**Impulsivity**	**1.45 (.018)**				246.23
DERS*		0.32 (<.001)	1.17 (.368)	**<.001**	224.10
CTQ		0.31 (<.001)	1.34 (.068)	.063	244.24
BIS-11		0.52 (<.001)	1.23 (.259)	.067	241.48
SCARE*		0.15 (.051)	1.30 (.123)	**<.001**	215.24
PHQ-9*		0.25 (<.001)	1.23 (.244)	**<.001**	210.26
**Emotional lability**	**2.13 (<.001)**				229.88
DERS*		0.67 (<.001)	1.43 (.092)	**.003**	222.03
CTQ		0.28 (<.001)	**2.03 (<.001)**	.148	229.61
BIS-11		0.41 (<.001)	**1.95 (<.001)**	.194	227.95
SCARE		0.61 (<.001)	**1.58 (.028)**	**.036**	212.64
PHQ-9*		0.60 (<.001)	1.43 (.075)	**<.001**	208.43
**Hostility**	**1.66 (.002)**				241.50
DERS*		0.42 (<.001)	1.27 (.181)	**<.001**	223.11
CTQ		0.35 (<.001)	**1.54 (.011)**	.129	240.80
BIS-11		0.31 (<.001)	**1.51 (.014)**	**.043**	236.47
SCARE		0.30 (<.001)	**1.47 (.038)**	**.001**	213.21
PHQ-9*		0.38 (<.001)	1.23 (.239)	**<.001**	210.24
**Perceptual dysregulation**	**1.89 (.004)**				241.57
DERS*		0.58 (<.001)	1.21 (.280)	**<.001**	223.72
CTQ		0.40 (<.001)	**1.65 (.035)**	.226	241.98
BIS-11		0.44 (<.001)	**1.58 (.042)**	.056	237.25
SCARE*		0.45 (<.001)	1.25 (.258)	**.001**	216.16
PHQ-9*		0.60 (<.001)	1.08 (.688)	**<.001**	211.47
**Distractibility**	**2.29 (<.001)**				226.85
DERS		0.54 (<.001)	**1.68 (.012)**	**.002**	218.29
CTQ		0.39 (<.001)	**2.18 (<.001)**	.370	228.01
BIS-11		0–58 (<.001)	**2.28 (<.001)**	.979	226.94
SCARE		0.30 (<.001)	**1.85 (.002)**	**.002**	207.02
PHQ-9		0.53 (<.001)	**1.57 (.032)**	**<.001**	206.85

*DERS* Difficulties in Emotion Regulation Scale, *CTQ* Childhood Trauma Questionnaire, *BIS* Barratt Impulsiveness Scale, *SCARE* Screen for Child Anxiety Related Emotional Disorders, *PHQ-9* Patient Health Questionnaire

^#^OR evaluated on the standardized variables. The reference group is NO-NSSI-BE

Unadjusted ORs refer to univariate logistic models results (first step of Baron and Kenny); adjusted ORs were carried out by multiple logistic models and refer to ORs of the personality traits adjusted for the clinical scale. The column of Spearman ρ coefficients represents the association between the traits and clinical scales (second step of Baron and Kenny); the column of adjusted ORs represents the association between personality traits and the group variable adjusted for the corresponding clinical scale (last step of Baron and Kenny)

* Highlights a mediation effect of clinical scale on the relation trait-group: i.e. when the clinical scale is introduced in the multiple logistic model, the ORs associated to traits (column Adjusted^#^ OR) become non-significant and the relative AIC decreases markedly

## Discussion

This study sought to explore the associations between ED, impulsivity, trauma experiences, depression, anxiety symptoms, personality traits and the occurrence of maladaptive behaviours.

### Maladaptive behaviours clusters and their clinical characteristics

Consistent with other findings among European adolescents [19, 39, 40], about 26% of participants reported to have engaged in NSSI at least once in their life, and 38% had at least one episode of binge eating. As expected, our findings by using MCA technique showed that adolescents with NSSI and/or BE behaviours fell into the same category and were different from other groups of students with other types of maladaptive behaviours or none. The high co-occurrence rate of these two self-damaging behaviours suggests that similar antecedents and mechanisms may be underlying [41, 42]. Several studies have found that people who engage in NSSI have a higher level of ED [40, 43, 44] supporting the notion that NSSI serves an emotion regulation function. In our study, adolescent students with higher depressive symptoms and ED scores were about 3 and 2.5 times more likely to belong to the NSSI-BE group. Moreover, higher scores in impulsivity and childhood trauma experiences increased the probability of belonging to the NSSI-BE group of 1.6 times. Previous studies have found that ED mediated the relationship between maltreatment exposure and self-harm among adolescents [45] and between emotional abuse and eating disorder symptoms [46]. Dvir and colleagues [47] argue that trauma exposure could impair the learning of emotion regulation skills that are potentially driven by interpersonal and attachment difficulties, and this, in turn, contributes to an increased risk of developing psychiatric symptoms during lifetime. Moreover, recent studies found that children who experienced maltreatment were significantly more likely to show borderline features than those who did not [48, 49].

### Personality traits predicting different cluster of maladaptive behaviours

Our findings confirmed that personality traits described as key BPD features are able to differentiate adolescents with NSSI-BE behaviours from their adolescent counterpart in the NO-NSSI-BE group, except for risk taking and suspiciousness. Separation anxiety, emotional lability, hostility and anxiousness are facets that are all included in the affective negative domain in the DSM-5 AMPD model; while impulsivity and distractibility are akin to the disinhibition domain [28]. In the current study, their associations with the NSSI-BE group are in line with previous studies in non-clinical young populations [16, 19, 20] using different dimensional models for personality disorder. In fact, these studies found that NSSI behaviours were associated with higher Neuroticism (akin to DSM-5 negative affectivity) and lower Conscientiousness (akin to DSM-5 disinhibition). Similarly, in relation to eating disorders, Brown and colleagues [50] found that Emotional stability (reverse of Neuroticism) and Conscientiousness predicted binge eating at 14 years and at 16 years of age. Furthermore, in line with a previous study [51], we found that depressivity and perceptual dysregulation were predictors of NSSI-BE behaviours. Compared to suspiciousness, that was not found significantly related to NSSI-BE in our study, perceptual dysregulation may better reflect dissociation and derealisation, that are described in criterion 9 of DSM-5 BPD diagnosis. There is sound evidence in support of the positive correlation between the severity of dissociation and the severity and frequency of self-harm in adolescents [52].

### Mediators of the relationship between personality traits and NSSI-BE behaviours group

Lastly, we have explored the relationship between self-reported personality traits and the NSSI-BE cluster after controlling for the effect of the clinical variables. In our study, ED, depression severity and, to a lesser extent, anxiety symptoms mediated the relationship with a variety of traits, mainly pertaining to the negative affectivity construct. Notably, some researchers proposed that deficits in ED increase the use of NSSI as an escape strategy in the presence of internalizing symptoms and internal emotional states perceived as aversive [53]. Another large-scale prospective study [50] found that lower emotional stability, and being identified as at risk for BPD in early childhood, predicted depressive symptoms, which in turn predicted binge eating and purging in adolescence. These are relevant results as psycho-educational interventions aimed to reduce ED, depressive and anxiety symptoms might buffer the impact of maladaptive traits on the occurrence of self-damaging behaviours such as NSSI and BE. Furthermore, in our study, the relationship between separation anxiety and distractibility traits and the occurrence of NSSI-BE was not influenced by none of the clinical variables; albeit in our study we did not include measures of BPD, we can argue that these two traits might represent an outstanding feature of the BPD diagnosis as reported elsewhere [29]. On the one hand, with regard to separation anxiety, this result is clinically compelling, since it is conceivable that higher sensitivity to abandonment is related to the disorganized attachment styles which BPD patients typically deal with [54]. On the other hand, with regards to distractibility, one interpretation is that it may be more related to cognitive control processes [5].

### Limitations

Some limitations of the current study should be acknowledged. First, the cross-sectional design of our study does not allow to test any causal relationships. Secondly, as our sample was predominantly female, same conclusions for male adolescents cannot be drawn. Recruitment was not heterogeneous for type of schools and our undergraduate student sample may exhibit better overall functioning than other community or clinical samples, hence findings may not generalize to other populations of adolescents. Thirdly, although our findings were largely consistent with previous studies, our study was exclusively focused on BPD-related traits. Further studies should explore other putative personality predictors of maladaptive behaviours in adolescence. Fourth, we assessed NSSI presence, but we did not use a scale to assess types and severity of NSSI. Finally, retrospective or longitudinal studies need to assess whether the traits observed in this study are factors of vulnerability for later BPD or if indeed they may be a generic risk factor for a variety of mental disorders associated to NSSI-BE behaviours.

## Conclusions

Our findings highlight the potential importance of focusing on depressive and anxiety symptoms and ED in school-based interventions aimed at preventing NSSI and BE behaviours. Recently, interventions focused on ED, such as acceptance-based emotion regulation group therapy and dialectical behaviour therapy [55, 56] adapted for adolescents have been delivered with encouraging findings in terms of reduction of risky behaviours. Adolescents with NSSI-BE and specific personality traits could represent a vulnerable group of adolescents, albeit the lack of a longitudinal observation does not allow to make inferences on the course of these aspects among those who presented heightened risk for the later emergence of BPD symptoms. Nevertheless, in a preventative prospective, as adolescents engaging in NSSI-BE showed higher levels of ED and vulnerability to depressive symptoms and anxiety, psycho-educational early interventions could be beneficial to potentially minimizing their risk of developing more severe forms of psychopathology.

## Supplementary Information

**Additional file 1: Figure S1** Correlation matrix of the clinical scales and personality traits in the overall sample

**Additional file 2:.**

## Data Availability

The dataset used during the current study is not publicly available but anonymized data are available from the corresponding author on reasonable request.

## Abbreviations

BPD: Borderline Personality Disorder
ED: Emotion Dysregulation
NSSI: Non-Suicidal Self-Injury
AMPD: Alternative DSM-5 Model for Personality Disorders
DERS: Difficulties in Emotion Regulation Scale
PID-5: Personality Inventory for DSM-5
BIS-11: Barratt Impulsiveness Scale-11
PHQ-9: Patient Health Questionnaire
SCARE: Screen for Child Anxiety Related Emotional Disorders
ERQ: Emotion Regulation Questionnaire
CR: Cognitive Reappraisal
ES: Expressive Suppression
MCA: Multiple Correspondence Analysis
