# Supplementary figures and images for: Maladaptive behaviours in adolescence and their associations with personality traits, emotion dysregulation and other clinical features in a sample of Italian students: a cross-sectional study
Source: Borderline Personal Disord Emot Dysregul. 2021 May 4;8:14. doi: 10.1186/s40479-021-00154-w (PMC8094601; doi:10.1186/s40479-021-00154-w)

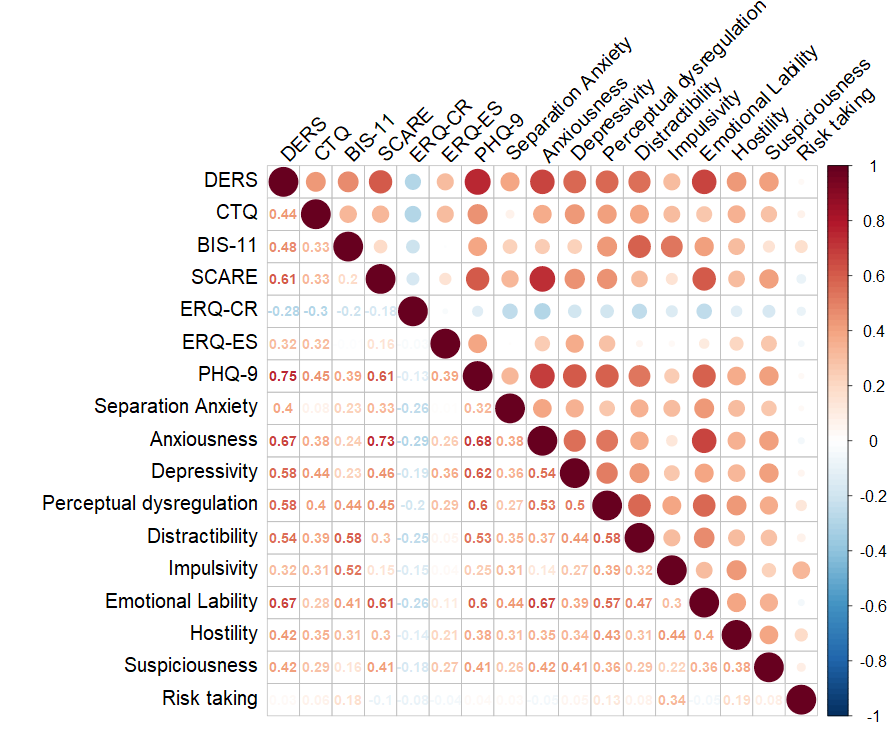

Supplement: Supplementary file 1 — Additional file 1: Figure S1 Correlation matrix of the clinical scales and personality traits in the overall sample [file 40479_2021_154_MOESM1_ESM.tiff]
