# Supplementary material for: Maladaptive behaviours in adolescence and their associations with personality traits, emotion dysregulation and other clinical features in a sample of Italian students: a cross-sectional study
Source: Borderline Personal Disord Emot Dysregul. 2021 May 4;8:14. doi: 10.1186/s40479-021-00154-w (PMC8094601; doi:10.1186/s40479-021-00154-w)
Supplement: Supplementary file 2 — Additional file 2:. [file 40479_2021_154_MOESM2_ESM.pdf]

## Supplementary Material

### Methods: Mediation model

We performed mediation analysis following the steps specified in Baron and Kenny (1986) performed by the Structural Equation Model -SEM- approach in order to take into account the variance-covariance structure of the variables involved in the mediation models. Moreover, to summarize all the SEM finding and for improving the readability of any mediation effect, the outputs of the mediation models were reported in terms of associations between personality traits and the student groups evaluated also in a multiple logistic model setting, by adjusting for the clinical scales. In fact, following Baron and Kenny, if the mediational model is correctly specified, the paths of X→ Y, X→M and M→ Y can be estimated by multiple (linear and logistic) regression.

We assessed one mediation model for each personality trait and for each clinical variable. In our case, the independent variables X are the personality traits, the dependent one is the group (NSSI-BE vs NO-NSSI-BE), and the mediator variables are the clinical scales.

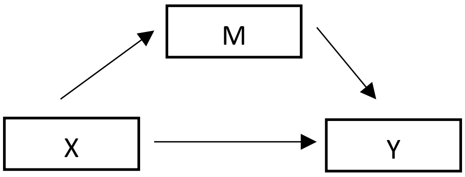


Baron and Kenny's Step 1:
The association between the personality traits (X) and the group variable (Y) has been checked (the results are shown in the second column of Table 3 – unadjusted OR)

$$Y = \beta_0 + \beta_1 X + \epsilon$$

Baron and Kenny's Step 2:
The association between the personality traits (X) and the clinical scales (M) has been checked (and reported, for simplicity of displaying, by Spearman $\rho$ in third column of Table 3)

Baron and Kenny's Step 3:
The association between the group variable (Y) and both the personality traits (X) and the clinical scales (M) has been analyzed. This step has been done to verify that:
(i) the mediator is significantly associated with the dependent variable, i.e. $\beta_2$coefficient of the equation below is significant (fifth column of Table 3 - Clinical scale p-value)
(ii) the association between the independent variable and the dependent one (evaluated in Step 1) is greatly reduced or no longer significant (fourth column of Table 3 - Adjusted OR)

$$Y = \beta_0 + \beta_1 X + \beta_2 M + \epsilon$$

Reference: Baron, R. M., & Kenny, D. A. (1986). The moderator-mediator variable distinction in social psychological research: Conceptual, strategic and statistical considerations. Journal of Personality and Social Psychology, 51, 1173-1182.
